# Understanding tuberculosis among people with tuberculosis through an educational film: a qualitative study

**DOI:** 10.1136/bmjopen-2025-103199

**Published:** 2025-08-19

**Authors:** Elin Economou Lundeberg, Olivia Biermann, Johanna Kuhlin, Asli Kulane, Kristi Sidney Annerstedt, Lina Davies Forsman

**Affiliations:** 1 Department of Infectious Diseases, Skåne University Hospital, Kristianstad, Sweden; 2 Department of Medicine, Division of Infectious Diseases, Karolinska Institute, Solna, Sweden; 3 Department of Global Public Health, Karolinska Institute, Stockholm, Sweden; 4 Department of Infectious Diseases, Karolinska University Hospital, Stockholm, Sweden

**Keywords:** Tuberculosis, QUALITATIVE RESEARCH, Patient-Centered Care, Medication Adherence, Health Education

## Abstract

**Abstract:**

**Introduction:**

Treatment of the two billion people with tuberculosis (TB) infection worldwide is crucial to prevent progression to TB disease and thereby prevent further transmission. However, TB is associated with fear and stigma, and knowledge gaps about TB disease are widespread, complicating adherence to treatment. As increasing knowledge about TB can reduce stigma and increase adherence to treatment, we developed an educational film about TB infection and disease. After showing the film to people with TB, our qualitative study aimed to evaluate the film and to explore perceptions, fears and possible knowledge gaps.

**Method:**

We conducted a qualitative study, with in-depth interviews (n=13), at two Infectious Disease Outpatient Departments in Sweden. Included research participants were adults with TB infection or TB disease. After informed consent, the participants watched the film, available in Swedish, English, Somali and Tigrinya. Subsequently, in-depth interviews, using a topic guide, were conducted, transcribed, and a reflexive thematic analysis was performed.

**Results:**

All participants considered the film to be a valuable addition to the written and oral information they had previously received. Identified themes included the perception of TB infection being a deadly, non-curable disease, and many feared being contagious. However, the film challenged these fears and increased the understanding of TB infection being treatable and non-infectious. Another theme revealed that TB-related stigma was experienced in encounters with healthcare professionals in Sweden.

**Conclusion:**

Our educational film was perceived to increase understanding about TB symptoms, transmission and treatment. Implementing the film in Infectious Disease Departments across Sweden may contribute to decreasing stigma and enhancing awareness of the importance of treatment adherence, an outcome that warrants further investigation post-implementation.

**STRENGTHS AND LIMITATIONS OF THIS STUDY**

- To the best of our knowledge, attempts to explore the introduction of educational information about TB in the format of a film have rarely been made.
- A demographically broad range of people with TB was included in the study, reflecting a diversity of different experiences regarding TB.
- The study used in-depth interviews providing abundant insights into the participants’ understanding of TB.
- Extensive discussions and revision of the coding process and themes identification involved the entire research team, allowing for better understanding and dependability of the data.
- A limitation was that the interviews were conducted in English or Swedish, and some nuances may have been lost if these languages were not the mother tongue of the participants.

## Introduction

 Tuberculosis (TB) is a major public health concern with approximately 1.3 million deaths yearly.^1^ Estimations conclude that around two billion people are infected with *Mycobacterium tuberculosis* bacteria and have a so-called TB infection.^2^ Individuals with TB infection (previously referred to as latent TB infection) lack clinical symptoms but are at risk of progressing to active TB disease.^3 4^ The WHO recommends screening for and treating TB infection, preventing progression to active TB disease. Preventive treatment of TB infection is preferable, as the preventive regimens are shorter and contain fewer drugs compared with TB disease treatment. From a community perspective, treating TB infection prevents future TB disease cases and thereby limits further TB transmission.^5^ Accordingly, prevention of active TB cases is an important part of the WHO End TB Strategy.^6^ However, adherence to treatment of TB infection can be as low as 47%, shown in a study from the US and Canada,^7^ or 76% in a Swedish setting.^8^

Barriers to adherence to treatment of TB infection include a lack of knowledge or misconceptions regarding TB. Misconceptions about TB have been identified in several qualitative interview studies: one misunderstanding is the mode of TB transmission, with many believing that TB disease spreads through sharing utensils, contaminated water or poor personal hygiene.^9^ In Kenya, only two out of 46 participants knew that TB was caused by a germ or bacteria.^10^ In a study in the USA, only 28% of patients receiving treatment against TB infection or disease knew what TB disease entailed.^11^ Other misconceptions include TB not being curable or, on the other hand, being easily treated by consuming alcohol.^11^ In a Swedish setting, one study^12^ illustrated a lack of understanding of the importance of treating TB infection. Misconceptions about TB infection can lead to an unnecessary fear of being contagious and infecting others.^13^

Another barrier to adherence to TB infection treatment is TB-related stigma. Stigma has been defined by Scambler^14^ as ‘a social process, experienced or anticipated, characterised by exclusion, rejection, blame or devaluation that results from experience, perception or reasonable anticipation of an adverse social judgement about a person or group’. Stigma can, for example, be anticipated (worry about judgment or devaluation because of a diagnosis) or experienced (behaviours directly endured by a person).^15^ In the case of TB, studies have shown that stigma involves shame of TB being associated with poverty and the risk of isolation from the community.^16^ Additionally, fear frequently arises in discussions about TB, including concerns about death, treatment of side effects and social repercussions such as unemployment.^16^ Shame, isolation and fears can hinder contact with healthcare and impede treatment adherence.^17^,^19^

Increasing knowledge about TB infection and disease can reduce stigma^9 20 21^ and thereby potentially increase treatment adherence. This association highlights the importance of education about TB. However, there are few studies addressing TB education and stigma reduction, particularly those with verbal information, which is preferred by many people with TB.^11 22^ Therefore, we developed an educational film, combining verbal information about TB infection and disease with visual aid, to explore experiences and perceptions regarding TB, aiming to understand possible knowledge gaps, stigma and misconceptions among people with TB.

## Methods

### Aim and design

We conducted a qualitative study based on in-depth interviews with people with TB infection or active TB disease. The aim of the study was two-fold: first, we aimed to explore perceptions, fears and possible knowledge gaps regarding TB disease and infection among people with TB, after having watched an educational film about TB. Second, we aimed to collate the patients’ views regarding the educational film, to improve it before a possible future implementation. This study is reported using the consolidated criteria for reporting qualitative research guidelines.^23^

### Theoretical underpinnings and framework

This study was informed by a constructionist epistemology.^24^ We approached participant narratives not as objective accounts of reality, but as lived experiences that are situated and shaped by social, cultural and personal factors. Our analysis was also guided by the conceptual model developed by Nuttall *et al*,^25^ which offers a framework for understanding patient engagement and behaviour in the context of TB care and specifically reducing TB-related stigma. We used this framework as a theoretical scaffold supporting our interpretation of how participants made sense of their TB experiences and the intervention, while remaining open to emergent themes beyond the scope of the model.

**Figure 1:**
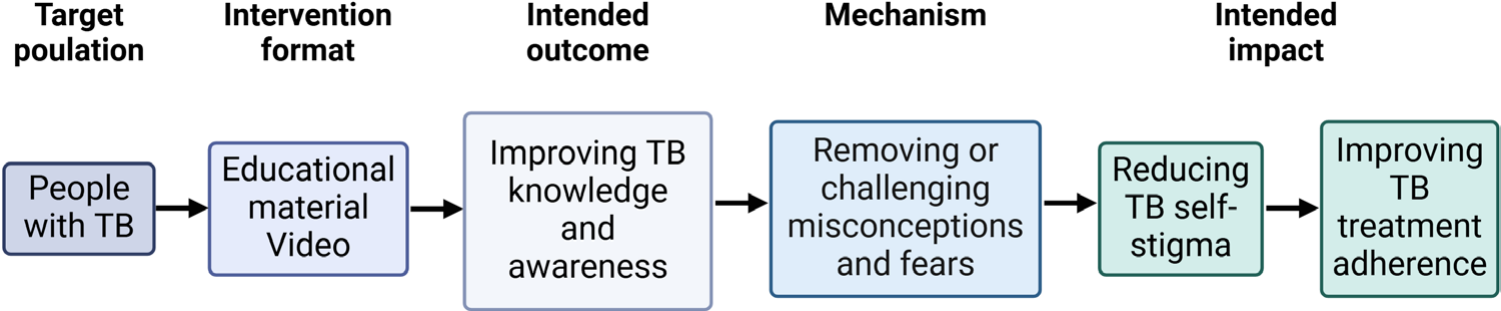
Framework adapted from Nuttall *et al*, Interventions pathways to reduce tuberculosis-related stigma: a literature review and conceptual framework; Infectious Diseases of Poverty; 2022. Created in BioRender. Economou Lundeberg, E. (2025) https://BioRender.com/w88i677. TB, tuberculosis.

A pathway in the framework includes a particular target population and intervention format, and thereafter suggests an intended outcome and impact of the intervention. In our study, we have adapted one of the pathways (figure 1) by making an educational film.

### Film production

The educational film covered symptoms of TB, how TB is transmitted, the difference between TB infection and TB disease and how TB can be treated. The film consisted of two parts: a short educational talk by an infectious disease specialist with large experience in working on both TB and global health (JK), combined with two publicly available educational films from different health organisations, KNCV and Vestre Viken, respectively. Permission was given by the two organisations to use the films, available in English, Tigrinya and Somali. The manuscript for JK’s part was developed by three infectious disease specialists with work and research experience in TB (EEL, LDF, JK), with input from other infectious disease physicians and nurses experienced in working with people with TB. The educational talk by JK was recorded in both English and Swedish. The different segments of the film, that is, the educational talk and the two films, were put together by a professional producer. The film was produced in four different languages (Swedish, English, Tigrinya and Somali) with a runtime ranging from 6 min and 30 s to 8 min, depending on the language. These languages were selected because the majority of people with TB disease over the past 8 years in Sweden were from Somalia and Eritrea.^26^

### Study setting and recruitment

The study took place at two outpatient infectious disease departments in Sweden: the Central Hospital of Kristianstad, Kristianstad and the Karolinska University Hospital, Solna, Stockholm. People with TB were approached during their planned appointment to one of these departments. In total, 18 people were asked to participate in the study, of whom 13 agreed to participate and gave their written consent. Five people declined participation, three due to lack of time in conjunction with their appointment and two due to lack of interest in participating. After their planned appointment with the physician or nurse, the participants watched the film in a separate room at the clinic, followed by an in-depth interview. The sampling was purposive with the aim of maximum variation of the demographic characteristics gender, age and country of birth. Three people were contacted by telephone by EEL after their appointment, due to a lack of time after their scheduled appointment. If interested in participating, a time for an interview at the clinic was planned in agreement with the person.

The sample size was estimated using the five dimensions of information power.^27^ The study had a narrow aim of exploring patients’ perceptions of a disease after having watched an educational film. Study participants were all highly specific to the target group, and together with an underlying theoretical framework, we originally estimated a sample size of around ten participants. However, the quality of the interview dialogues varied, and as an exploratory analytic approach was used, we decided to conduct an additional three interviews.

### Study participants

Eligible participants were adults newly diagnosed with TB infection or disease, attending their first or second visit to the infectious disease outpatient department. A total of 13 participants were included from June 2022 to October 2023, one diagnosed with TB disease and the rest with TB infection. The age range of the participants was 18–78 years. Participants were born in nine different countries and spoke at least one of the languages in which the film was available. Socio-demographic characteristics are presented in table 1.

**Table 1:** Characteristics of people with tuberculosis participating in the study

Characteristic	Number
Self-reported gender	
Female	9
Male	4
Highest completed education	
University	8
High school	2
Elementary school	2
Continent of origin	
Europe	6
Africa	5
Asia	1
South America	1

### Data collection and analysis

The interviews were conducted by EEL (nine), JK (three) and LDF (one) in a separate room at the infectious disease outpatient departments of the two hospitals. All three interviewers were infectious disease specialists working with people with TB regularly. The interviews were conducted using a topic guide available in Swedish and English and took between six and 36 min. As multiple interviewers conducted the interviews, all three interviewers were involved in the development of the topic guide and discussed interview techniques before the first interview. During the interviews, the term ‘latent tuberculosis’ was used instead of ‘TB infection’, as it better matches Swedish terminology. The topic guide with open-ended questions was developed by EEL, JK and LDF and the experienced qualitative researcher KSA (online supplemental appendix 1). The topic guide was further revised by EEL and KSA in the middle of the study period (online supplemental appendix 2). The revision was initiated by the interviewers as some questions did not allow reflexive responses from the participants. Rephrasing some questions allowed for a better approach to asking the question, for example, expanding the question ‘What is the general perception of TB in your community?’ to include ‘What do you think about the disease?’. The guide covered all seven components of the theoretical framework of acceptability of healthcare interventions, developed by Sekhon *et al*.^28^ Ten interviews were conducted in Swedish and three in English. The interviews were audio recorded and later transcribed verbatim by LDF (one) and EEL (twelve). The quotes in Swedish have been translated by a native English speaker, being bi-lingual, and grammatical errors have been corrected.

Reflexive thematic analysis, according to Braun and Clarke,^29^ with further guidance from Nowell *et al*,^30^ was performed. The coding was done in NVivo V.14 (Lumivero). The first two interviews were coded by both OB and EEL; the results were compared and the codes were revised. The remaining coding was performed by EEL. A list of codes was created, and all data were checked repeatedly to revise previous coding. From the codes, categories were inductively composed from patterns of meaning in the data and discussed with LDF and OB. Thereafter, categories were diagrammed into a concept map to find possible associations between the categories and between their meanings. The categories were also related to the framework by Nuttall *et al*,^25^ by comparing the categories to the mechanisms of stigma reduction proposed by the framework (figure 1). Themes were identified from associations between the categories and their meaning, and in relation to the framework. Phases 4 and 5 (reviewing and defining themes) of Braun and Clarke’s thematic analysis process were discussed and revised by all co-authors. Examples of the thematic analysis process are presented in table 2.

**Table 2:** Examples of the thematic analysis process

Example quote	Code	Category	Sub-theme	Theme
‘In my country, people don’t actually talk about such a disease. If somebody has this disease, you don’t tell others (…). Or maybe he’s afraid that people believe that they have this kind of disease, so people don’t actually talk about those issues. They are hiding by the way’. (Interviewee #9)	Stigma	Perceptions, fear and stigma	Fear of isolation	TB is not talked about
‘Before now, I thought of tuberculosis (TB) as this very infectious disease; I never knew it could be treated. I just watched it on the video now and was like ‘wow’. I was amazed’ (Interviewee #1)	Adding knowledge	Understanding and knowledge gap	TB is treatable	Film challenged fears of TB being deadly and always contagious

### Ethics

The study was approved by the Swedish Ethical Review Authority (numbers 2022-00722-01 and 2022-04215-02), and participants’ data were handled in accordance with the general data protection regulation. All participants were given oral and written information about the study, including information that they could withdraw at any point. Study participants provided written informed consent.

### Patient involvement

As part of the in-depth interviews, the patients were asked how we could improve the content of the educational film. They were also asked about their opinions regarding a future implementation process of the film, for example, at which sites the film could be presented and the timing of film presentation in the process of TB care management. These suggestions will be taken into consideration for the forthcoming development of the film and the planned implementation process of the education film throughout Sweden.

## Results

**Figure 2:**
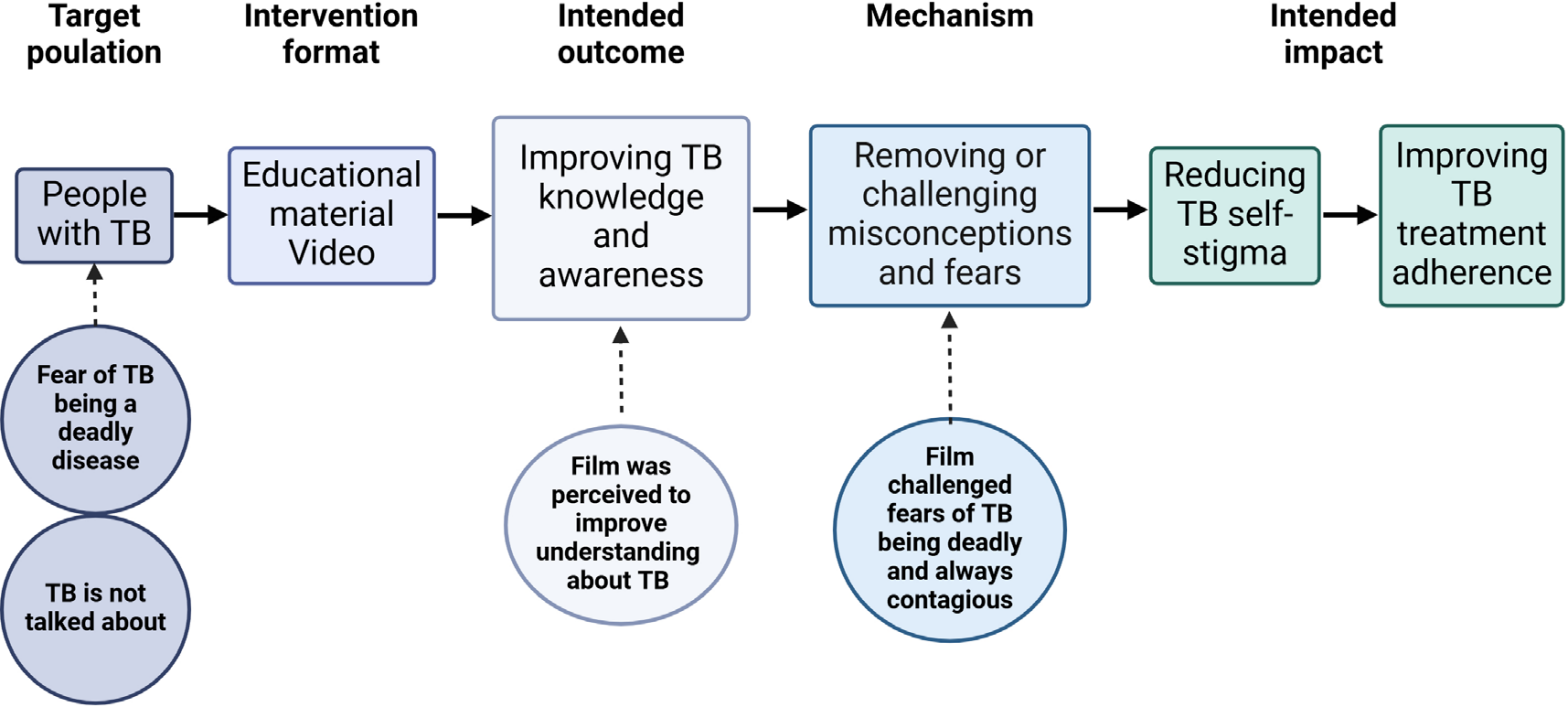
Identified themes shown in relation to framework adapted from Nuttall *et al*, Interventions pathways to reduce tuberculosis-related stigma: a literature review and conceptual framework; Infectious Diseases of Poverty; 2022. Created in BioRender. Economou Lundeberg, E. (2025) https://BioRender.com/m47m516. TB, tuberculosis.

We identified four themes and seven sub-themes from the data, summarised in table 3 and shown in relation to the conceptual framework^25^ in figure 2. The themes are presented by first describing participants’ perceptions of TB, that is, themes 1 and 2. Thereafter follow themes 3 and 4, in accordance with the framework’s intended outcome (improving TB knowledge and awareness) and mechanism (removing or challenging misconceptions or fears) of an intervention. Finally, the participants’ suggestions regarding possible improvements to the film before implementation are described.

**Table 3:** Themes and sub-themes were identified during thematic analysis

No.	Theme	No.	Subtheme
1	Fear of TB being a deadly disease	1.1	Fear of infecting others
		1.2	Family and friends are worried about your TB diagnosis
2	TB is not talked about	2.1	Fear of isolation
		2.2	Sharing TB diagnosis induces negative reactions, including from Swedish healthcare professionals
3	Film was perceived to improve the understanding of TB	3.1	Latent TB infection is not contagious
		3.2	Rephrasing ‘latent infection’ would further facilitate the understanding of TB disease stages
4	Film challenged fears of TB being deadly and always contagious	4.1	TB is treatable

TB, tuberculosis.

### Fear of TB being a deadly disease

Participants expressed several fears related to TB, including fear of TB being a deadly disease, infecting others and family worrying about their TB diagnosis. The idea of TB being a deadly disease was a common reflection. For example, one participant stated:

So, the first thought when they said it is tuberculosis was: ‘Okay, I will die by like vomiting blood’. (interviewee #4, female, 40s)

Several participants’ experiences of TB were in relation to relatives having TB many decades ago. One participant also compared with the past and stated that TB was more of a deadly disease a century ago – *‘because there is treatment (now) and there are ways to be rid of it. But in the past, it was very stressful and nerve-racking because many people died from this disease*’ (interviewee #8, female, 50s). Most participants’ largest fear was to infect other people with TB and described how they told others to keep away from them and even used special plates and utensils for visitors to avoid transmitting the disease. Parents, especially mothers, expressed concern about infecting their children and saw this as their hardest issue to deal with when receiving the diagnosis:

Yes (I am thinking about the diagnosis), because I’ve got children, small, seven months, so if I get active (TB), I don’t want to infect (them). (interviewee #10, female, 40s)

Another concern expressed by some was that their friends and family would worry about their TB diagnosis. Some participants even avoided telling their relatives to spare them from worrying—*‘I’ve been thinking that maybe it’s not something they need to know, and I mean, I’m not at risk of spreading the disease. But just to not scare them either’* (interviewee #12, male, 30s).

### TB is not talked about

The study participants described negative experiences associated with having TB, with several persons admitting that they had never talked about it with anyone else:

No, we never talk about it. I have never talked about it and never met anyone with it. (interviewee #5, female, 30s)

Reasons not to tell others or not to talk about TB included lack of knowledge of the disease, fear of the unknown and of isolation from society. Participants explained that sharing their TB diagnosis might deter others from visiting them out of fear and even gave examples of physical assaults against people living with TB. Some participants also described that people with TB might hide, fearing that others know about their disease.

In my country, people they don’t actually talk about this kind of disease. If somebody has this disease, you don’t tell others (…). Or maybe he’s afraid that people believe that they have this kind of disease, so people don’t actually talk about those issues. They are hiding (…). People will run away from them. Sometimes people do insults to them. (interviewee #9, female, 40s)

Other reasons for not disclosing their TB diagnosis included a fear of triggering negative reactions, such as being avoided by others. Two participants highlighted that negative reactions existed among Swedish healthcare personnel:

I feel that here in Sweden, they are more afraid. When you tell them that I have latent TB—‘oh, I shouldn’t have contact with you’ and stuff like that. (…) They (health care personnel) should be more used to the disease. (…) Because they shouldn’t be so afraid of ‘oh you have TB’. (interviewee #8, female, 50s)

However, other participants stated that their TB diagnosis was not a secret and that they felt comfortable telling friends and family about their diagnosis. One participant also had thoughts on how to reduce TB-related stigma and awareness of TB-related stigma is also demonstrated by one participant*—‘you start (talking) in a friendly way, so you don’t try to stigmatise them because it’s not really fair, and you don’t make them feel fear of themselves*’ (interviewee #1, female, 30s).

### The film was perceived to improve the understanding of TB

All participants thought the film improved their understanding of TB, in particular in terms of TB symptoms, transmission and treatment. Primarily, after having watched the film, the participants described an increased awareness of TB symptoms. Beforehand, many participants had an idea of TB being exclusively a pulmonary disease, but after the film, they displayed knowledge of extra-pulmonary TB symptoms, such as enlarged lymph nodes and back pain. Weight loss and night sweats were also mentioned as new information.

Before, I knew only the lung TB, and now I know different kinds of TB with the skeleton, the knots and the lungs. (…) I was not aware that sweating is a symptom of TB. I was only aware of the coughing, only the coughing. (interviewee #4, female, 40s)

Furthermore, many participants demonstrated an increased awareness of how TB was transmitted after having watched the film. They were surprised by the information that only active lung TB was contagious—*‘that it is only from the lungs that it can infect. I didn’t know that*’ (interviewee #7, male, 30s).

The concept of TB infection was also new to many participants. In some of the participants’ countries of birth, TB infection is neither tested for or treated, and many participants mentioned that they had never heard of TB infection before. Many thought of TB as one single disease entity*—‘That there are two different types… I didn’t know that, thought there was only one. That you have tuberculosis, that’s it*’ (interviewee #2, female, 20s). One participant said that she had not known why she had to take the preventive treatment before, but now, after seeing the film, she understands why she had taken it:

Now I get why. But before, I didn’t really know. Because they told me: ‘oh, you have this and this and you need medicine’. And that’s it. So, it would have been a little better if I had got a better understanding of what medicine I was going to take for how long exactly. (interviewee #11, female, 20s)

Even though the concept was new to many of the participants, several appreciated becoming aware of their TB infection. The appreciation was two-fold: being able to get rid of the bacteria before the infection developed into active TB disease, and being aware of the symptoms of active TB:

I’m more risk-aware that latent tuberculosis can turn into active tuberculosis. And then you know the symptoms in advance. (interviewee #7, male, 30s)

In line with the participants’ acquired awareness of TB infection, the knowledge of TB infection not being contagious was a relief for many*—‘I realised it (TB) wouldn’t infect anyone else (…) It feels good*’ (interviewee #5, female, 30s). Many participants also reflected on the word ‘latent’ when dealing with TB infection without any symptoms. They thought the word ‘latent’ was hard to understand and even sounded harsh*—‘Latent is, also can feel that just that word is a bit of a risk somehow, risky. (…) Explosive*’ (interviewee #6, female, 50s).

A consequence was also that the whole concept of latent infection became confusing. Several participants suggested changing the word ‘latent’ into ‘sleeping’ to make the concept easier to comprehend:

If they’d said to me, ‘you have a sleeping tuberculosis’—I would have known what it was. Latent can be a hard word, but now I understand what it is, but it’s not that everybody knows. And most patients actually don’t know. (interviewee #11, female, 20s)

The participants also expressed a new understanding of TB treatment, both against TB infection and disease. The idea of preventive treatment was easier to grasp, and why treatment is recommended for TB infection. Several participants mentioned the value of knowing that treatment is for free*—‘They (the film) tell you that medicines are free, without money, just that you’re willing to take the tablets*’ (interviewee #13, male, 30s).

### The film challenged fears of TB being deadly and always contagious

Fears described by many participants, such as fear of TB being a deadly disease and fear of infecting others, were challenged by the film. Knowing more about how TB is treated and how it is transmitted decreased the anxiety that participants may have felt before watching the film—*‘before I thought of it as this deadly disease. So, I have seen (the film) now and the treatment, the way it goes, I think, I feel more relaxed*’ (interviewee #1, female, 30s). The film conveyed the intended message of TB being an infection or disease that is treatable, which was reassuring to many participants:

It’s made pretty clear that this (infection) is not a problem, we will manage it. So, I think that was good. (interviewee #3, male, 80s)

Some participants reflected on the added knowledge from the film that made them feel less guilty*—‘So at least now I don’t feel bad because I don’t have it (TB disease) and then it can’t be transmitted, so I don’t feel bad anymore*’ (interviewee #1, female, 30s). The added knowledge also made some participants feel safer. This feeling was also demonstrated by people with a medical education and previous knowledge about TB, of which one expressed:

I actually felt a little bit more secure even though I knew about it before. (interviewee #12, male, 30s)

After viewing the film, participants primarily expressed two key concerns. First, many questioned the origin of their TB infection, wondering where and how they may have been exposed. Additionally, some participants raised doubts regarding the efficacy of treatment and wondered *‘if you really get well after treatment?*’ (interviewee #6, female, 50s).

### Participants’ opinions and suggestions regarding improvements to the film

In general, the film was well received by the participants. They expressed that the information was easy to grasp, and it clarified a lot of previous thoughts and questions*—‘Now I have an idea about what tuberculosis is … before I hadn’t any idea, so now, I know more’* (interviewee #9, female, 40s). Participants thought the language of the film was easy to understand, and they were able to concentrate throughout the entire duration of the film. Participants with Somali or Tigrinya as their mother tongue confirmed a correct translation of the film.

However, some participants voiced negative feedback regarding the film as well. They felt it was too slow and repetitive. Improvements were suggested, for example, more statistics about treatment success and reinfection rates, treatment options and side effects. Other suggestions reflected the participants’ remaining thoughts about where they had acquired the infection and thus wanted to know more about TB epidemiology and country-specific prevalence*—‘point out a little bit more specifically where in the world it is most commonly found*’ (interviewee #7, male, 30s).

The majority of participants thought that the best location to watch the film was right before the visit to the physician or nurse treating TB. Watching the film at home before the visit could induce fears and questions, which would remain unanswered for a longer period of time. On the other hand, some participants thought that the film should be made available to the public to increase the general knowledge of TB in the Swedish population. Furthermore, one participant suggested that we should implement the film on a broader scale, including primary healthcare facilities and at the national education programme ‘Swedish for immigrants’.

## Discussion

Our study introduced an educational film about TB, after which participants’ perceptions, fears and possible knowledge gaps were explored. We derived four major themes: the participants expressed concerns of TB being a lethal, highly infectious disease and something that is not talked about. However, the film was perceived to improve the understanding of TB symptoms, transmission and treatment and challenged the fears of TB being deadly and always contagious. Furthermore, participants’ suggestions for changes to the film can improve the film before implementation.

Our findings resonate with the theoretical framework proposed by Nuttall *et al*,^25^ particularly regarding improving awareness of TB and challenging fears and misconceptions regarding the disease. Participants’ experiences underscore how educational material can enhance understanding of TB, affirming the applicability of this framework in comprehending TB care engagement in our context. The first theme derived in our study, the fear of TB being a deadly disease, relates to our study’s target population being people with TB in the framework model (figure 2). The fear of TB being a deadly disease has been found in previous studies of people living with TB infection or disease.^13 31 32^ Fear of being contagious is also common, especially in regard to infecting children and others in the household.^13 33^ As TB is largely a curable disease, the participant’s perception of TB being deadly and very contagious leads to unnecessary worrying and anxiety. Increasing understanding of the disease, as suggested by Nuttall *et al* as an intended outcome of the framework, can challenge misconceptions and lessen fears related to TB. This is further demonstrated by our fourth theme, that the film challenged fears of TB being deadly and always contagious, constituting a mechanism to reduce stigma (figure 2). The participants described feeling safer, more relaxed and secure after understanding that TB is treatable, how the treatment is taken and that TB infection is non-contagious.

Participants described situations including both anticipated and enacted stigma,^15^ as reflected in our second theme. Anticipated stigma was characterised by a reluctance to disclose their TB diagnosis driven by the fear of the unknown, fear of isolation from society and even fear of violence. These narratives were especially prominent among participants born in high-endemic countries. Similar experiences of anticipated stigma are reported from high-endemic areas^34^ and portrayed by immigrants living in low-incidence settings.^9 12^

The consequences of stigma include reduced health-seeking behaviour and treatment adherence. This is supported by a quantitative evaluation of treatment completion rates of TB infection in Sweden, which showed that individuals of Somali origin had nearly four times higher risk of non-completion compared with people from European countries.^8^ Examples of enacted stigma were reported in relation to Swedish healthcare personnel. Participants described how a lack of knowledge about TB symptoms hindered contact with healthcare, and fear of TB was sometimes expressed through discriminatory or distancing behaviours. Other studies have reported TB-related stigma among healthcare professionals,^35 36^ highlighting persistent knowledge gaps about TB transmission and disease progression.

Increasing knowledge and awareness of TB among Swedish healthcare personnel may, according to our study, reduce enacted stigma experienced by people with TB and contribute to decreased patient delay in seeking healthcare. In the framework by Nuttall *et al*, one intervention pathway to reduce stigma targets TB-specific healthcare workers. However, our findings extend the framework by highlighting the need to engage also non-TB healthcare staff through educational interventions, as they too may influence stigma experiences and access to care for people with TB. Although structural racism has not been widely studied in the Swedish healthcare context, it has been acknowledged in the literature and by authorities such as the Equality Ombudsman,^37^ and may intersect with TB-related stigma.

Our study’s third theme was that the educational film was perceived by all participants to increase their understanding of TB symptoms, transmission and treatment. Implications of the increased understanding, after having watched the film, included less fear of infecting others, less guilt of having the infection, optimism of having a curable condition and higher motivation to continue treatment of TB infection. These findings suggest that an educational film can fulfil the intended outcome of improving TB knowledge and awareness, also in line with the theoretical framework (figure 2). One study based on observations in TB clinics in El Salvador found that introducing educational visual material improved patient understanding of what TB is, how it is transmitted and how it is treated, and adherence to treatment.^38^ However, there remains a lack of high-quality studies to explore the associations between patient education and increased adherence^22^ or between educational interventions and decreased stigma.^21 25^ A study evaluating stigma as the outcome could preferably use a validated stigma measurement tool,^15 39^ as recommended by Nuttall *et al*.^25^

Our study also collated the participants’ views regarding the film. Valuable feedback was given, including that many participants had remaining thoughts about where they had acquired the infection. Thus, suggestions for improvement included more information about TB epidemiology and country-specific prevalence. Statistics about treatment success, reinfection rates and possible side effects could also be added before implementation. Co-designing patient education interventions with the presumed target group has the advantage of providing information easily apprehended by the target group in question.^40^ Furthermore, the educational material consists of information valuable to patients, including topics that healthcare professionals might not have thought to be important.^40 41^ Improving our film according to our participants’ suggestions is an important step before starting the implementation process.

### Strengths and limitations

We have introduced an educational film about TB and interviewed a demographically broad range of people with TB about their experiences regarding TB, after having watched the film. Attempts to explore the introduction of visual information about TB in a film format have rarely been done. Another strength is the study authors, including experienced researchers in relation to treating people with TB and to qualitative research, respectively. Extensive discussions and revisions of the coding process and themes identification involved the entire research team, allowing for a better understanding and dependability of the data. A wide range of people with TB were asked to participate, but well-educated people agreed to participate to a higher degree than asylum seekers, resulting in a slightly less varied study sample. This trend can, however, be reflected in the fear of authorities and concerns about revealing personal identification data, as described in previous Swedish studies.^12^

As a research team, we maintained reflexive awareness of the sample’s heterogeneity, especially the inclusion of both individuals with TB infection and those with active TB disease. This awareness informed our coding and theme development, encouraging us to question whether our interpretations were shared or contingent on participants’ experiences with TB infection or disease. The team discussed our assumptions about the potential influence of clinical background on perceptions of the intervention, and how this might have influenced our interpretation of the data. These reflexive practices supported a more nuanced and transparent analysis, which was crucial given the heterogeneity in our sample.

A limitation was that the film was only available in four different languages, and the interviews were conducted in English and Swedish. Even though the participants spoke one of the interview languages, some nuances may have been lost. Another reflection included that JK was in the film, presenting information about TB. She also performed three of the interviews and possibly introduced acquiescence bias affecting the responses of these interviewees. Moreover, in some cases, the interviewers were treating physicians to the participants, which could have instituted a power difference. However, we made clear that the views of the participants were completely anonymous and did not affect their treatment. Participants did express negative reactions towards the film, showing that the possible power difference did not affect the results. Also, by posing questions about stigma, we could have insinuated the relationship between TB and stigma, but some participants were clear about having no fears or stigma. The inclusion of only one participant with TB disease limited the depth of insight into how individuals with differing clinical presentations may interpret the intervention. While this limits transferability to all populations affected by TB, we believe the diversity of experiences within the infection group still provides meaningful insight into how the intervention may be received and adapted in practice.

## Conclusion

In our study, the educational film was perceived by people with TB to increase their understanding of TB symptoms, transmission routes and treatment. Increasing understanding of TB constitutes the intended outcome of intervention pathways to reduce stigma, as suggested by Nuttall *et al*’s theoretical framework.^25^ The film also challenged misconceptions and fears related to TB, described as a mechanism of stigma reduction. Changes to the film, suggested by the participants, can further improve the film. Through a better understanding of TB, we anticipate a decrease in stigma and a potential increase in adherence to treatment,^25^ effects that should be further investigated after implementation of the film.

## Supplementary material

10.1136/bmjopen-2025-103199 online supplemental file 1

10.1136/bmjopen-2025-103199 online supplemental file 2

## Data Availability

Data are available upon reasonable request.

## References

[1] World Health Organization (2024). Global tuberculosis report 2024.

[2] Cohen A, Mathiasen VD, Schön T (2019). The global prevalence of latent tuberculosis: a systematic review and meta-analysis. Eur Respir J.

[3] World Health Organization (2018). Latent Tuberculosis Infection: Updated and Consolidated Guidelines for Programmatic Management.

[4] World Health Organization (2024). WHO Consolidated Guidelines on Tuberculosis. Module 1: Prevention – Tuberculosis Preventive Treatment.

[5] Kim HW, Kim JS (2018). Treatment of Latent Tuberculosis Infection and Its Clinical Efficacy. Tuberc Respir Dis (Seoul).

[6] World Health Organization (2015). The End TB Strategy: Global Strategy and Targets for Tuberculosis Prevention, Care and Control After.

[7] Horsburgh CR, Goldberg S, Bethel J (2010). Latent TB infection treatment acceptance and completion in the United States and Canada. Chest.

[8] Kan B, Kalin M, Bruchfeld J (2013). Completing treatment for latent tuberculosis: patient background matters. Int J Tuberc Lung Dis.

[9] Spruijt I, Haile DT, van den Hof S (2020). Knowledge, attitudes, beliefs, and stigma related to latent tuberculosis infection: a qualitative study among Eritreans in the Netherlands. BMC Public Health.

[10] Mbuthia GW, Olungah CO, Ondicho TG (2018). Knowledge and perceptions of tuberculosis among patients in a pastoralist community in Kenya: a qualitative study. Pan Afr Med J.

[11] Royce RA, Colson PW, Woodsong C (2017). Tuberculosis Knowledge, Awareness, and Stigma Among African-Americans in Three Southeastern Counties in the USA: a Qualitative Study of Community Perspectives. J Racial Ethn Health Disparities.

[12] Kulane A, Ahlberg BM, Berggren I (2010). “It is more than the issue of taking tablets”: the interplay between migration policies and TB control in Sweden. Health Policy.

[13] Shedrawy J, Jansson L, Röhl I (2019). Quality of life of patients on treatment for latent tuberculosis infection: a mixed-method study in Stockholm, Sweden. Health Qual Life Outcomes.

[14] Scambler G (2009). Health-related stigma. Sociol Health Illn.

[15] Challenge TB (2018). TB Stigma Measurement Guidance.

[16] Juniarti N, Evans D (2011). A qualitative review: the stigma of tuberculosis. J Clin Nurs.

[17] Gebreweld FH, Kifle MM, Gebremicheal FE (2018). Factors influencing adherence to tuberculosis treatment in Asmara, Eritrea: a qualitative study. J Health Popul Nutr.

[18] Courtwright A, Turner AN (2010). Tuberculosis and stigmatization: pathways and interventions. Public Health Rep.

[19] Teo AKJ, Ork C, Eng S (2020). Determinants of delayed diagnosis and treatment of tuberculosis in Cambodia: a mixed-methods study. Infect Dis Poverty.

[20] Yin X, Yan S, Tong Y (2018). Status of tuberculosis-related stigma and associated factors: a cross-sectional study in central China. Trop Med Int Health.

[21] Sommerland N, Wouters E, Mitchell EMH (2017). Evidence-based interventions to reduce tuberculosis stigma: a systematic review. Int J Tuberc Lung Dis.

[22] M’imunya JM, Kredo T, Volmink J (2012). Patient education and counselling for promoting adherence to treatment for tuberculosis. Cochrane Database Syst Rev.

[23] Tong A, Sainsbury P, Craig J (2007). Consolidated criteria for reporting qualitative research (COREQ): a 32-item checklist for interviews and focus groups. Int J Qual Health Care.

[24] Green JT (2018). N. Qualitative Methods for Health Research.

[25] Nuttall C, Fuady A, Nuttall H (2022). Interventions pathways to reduce tuberculosis-related stigma: a literature review and conceptual framework. Infect Dis Poverty.

[26] Folkhälsomyndigheten (2024). Tuberkulos - sjukdomsstatistik. https://www.folkhalsomyndigheten.se/folkhalsorapportering-statistik/statistik-a-o/sjukdomsstatistik/tuberkulos/?tab=tab-country.

[27] Malterud K, Siersma VD, Guassora AD (2016). Sample Size in Qualitative Interview Studies: Guided by Information Power. Qual Health Res.

[28] Sekhon M, Cartwright M, Francis JJ (2017). Acceptability of healthcare interventions: an overview of reviews and development of a theoretical framework. BMC Health Serv Res.

[29] Braun V, Clarke V (2022). Thematic Analysis: A Practical Guide.

[30] Nowell LS, Norris JM, White DE (2017). Thematic Analysis:Striving to Meet the Trustworthiness Criteria. Int J Qual Methods.

[31] Long NH, Johansson E, Diwan VK (2001). Fear and social isolation as consequences of tuberculosis in VietNam: a gender analysis. Health Policy.

[32] Karim F, Johansson E, Diwan VK (2011). Community perceptions of tuberculosis: A qualitative exploration from a gender perspective. Public Health.

[33] Jansson L, Shedrawy J, Lönnroth K (2020). Latent tuberculosis in pregnant women: a patient perspective. Int J Tuberc Lung Dis.

[34] Tadesse S (2016). Stigma against Tuberculosis Patients in Addis Ababa, Ethiopia. PLoS ONE.

[35] Coreil J, Lauzardo M, Heurtelou M (2012). Anticipated tuberculosis stigma among health professionals and Haitian patients in South Florida. J Health Care Poor Underserved.

[36] Sima BT, Belachew T, Abebe F (2019). Health care providers’ knowledge, attitude and perceived stigma regarding tuberculosis in a pastoralist community in Ethiopia: a cross-sectional study. BMC Health Serv Res.

[37] Bradby H, Thapar-Björkert S, Hamed S (2019). Undoing the unspeakable: researching racism in Swedish healthcare using a participatory process to build dialogue. Health Res Policy Sys.

[38] Wilson JW, Ramos JG, Castillo F (2016). Tuberculosis patient and family education through videography in El Salvador. J Clin Tuberc Other Mycobact Dis.

[39] Van Rie A, Sengupta S, Pungrassami P (2008). Measuring stigma associated with tuberculosis and HIV/AIDS in southern Thailand: exploratory and confirmatory factor analyses of two new scales. Trop Med Int Health.

[40] Hyatt A, Morkunas B, Davey D (2021). Co-design and development of online video resources about immunotherapy with patients and their family. Patient Educ Couns.

[41] Low JK, Crawford K, Manias E (2016). A compilation of consumers’ stories: the development of a video to enhance medication adherence in newly transplanted kidney recipients. J Adv Nurs.

