# Supplementary material for: Understanding tuberculosis among people with tuberculosis through an educational film: a qualitative study
Source: BMJ Open. 2025 Aug 19;15(8):e103199. doi: 10.1136/bmjopen-2025-103199 (PMC12366580; doi:10.1136/bmjopen-2025-103199)
Supplement: online supplemental file 1 [file bmjopen-15-8-s001.docx]

**Appendix 1. Topic guide EduTB**

1. Information about the study and its purpose. Informed consent (Anonymously, confidential, can end at any time). Interview will be recorded.
2. Basic information about the interviewee.
   1. Name:
   2. Age:
   3. Country of origin:
   4. Year of arrival to Sweden (if applicable):
   5. Mother tongue:
   6. Previous education:
3. Semi-structured interview:

**Knowledge gaps**

- What is your first thought after watching the film?
- Were you surprised by any of the information in the film and if so, what?
- What sort of questions does the film raise regarding TB disease?
- Describe your previous experience of TB disease.
- Which aspects of TB disease would you like to know more about?
- In what ways have your understanding of TB disease changed after this film?

**Perceptions**

- How did you feel after watching the film?
- Was there anything in the film that made you scared or worried? If so, what was that and in what way?
- “You don’t have a disease, but you need treatment” – what are your thoughts and understanding about this quotation?
- What is the general perception of TB in your community? (And you personally?)
- In your community, how is a person who has LTBI usually thought of?
- Will you feel comfortable to discuss your TB/latent TB diagnose and preventive treatment with other people in your community?

**Evaluation of the film**

- What changes to this film would you suggest in order to improve its message?
- What other means of information would you suggest to increase knowledge of TB?
- How do you usually get information about health issues and diseases?

“Tell me more…” “What do you mean?…” Can you give some examples?…”

4. End of interview:

Do you have any questions to ask?

Ensure that the interviewee has full name and contact details of interviewer.

Thank the interviewee for their time!
