# Supplementary material for: Understanding tuberculosis among people with tuberculosis through an educational film: a qualitative study
Source: BMJ Open. 2025 Aug 19;15(8):e103199. doi: 10.1136/bmjopen-2025-103199 (PMC12366580; doi:10.1136/bmjopen-2025-103199)
Supplement: online supplemental file 2 [file bmjopen-15-8-s002.docx]

**Interview guide EduTB**

1. Information about the study and its purpose. Informed consent (Anonymously, confidential, can end at any time). Interview will be recorded.
2. Basic information about the interviewee.
   1. Name:
   2. Age:
   3. Country of origin:
   4. Year of arrival to Sweden (if applicable):
   5. Mother tongue:
   6. Previous education:
3. Semi-structured interview:

**Knowledge gaps**

- What is your first thought after watching the film?
- In what ways were you surprised by any of the information in the film? How does this make you feel?
- What sort of questions does the film raise regarding TB disease?
- Describe your previous experience of TB disease. What do you think of TB disease?
- Which aspects of TB disease would you like to know more about?
- In what ways have your understanding of TB disease changed after this film?

**Perceptions**

- How did you feel after watching the film?
- Was there anything in the film that made you scared or worried? If so, what made you feel this way and how did you feel? Have you yourself felt like this?
- Explain again differences between active and latent tuberculosis “You don’t have a disease, but you need treatment” – what are your thoughts about this quotation? How is the sleeping kind treated in your country?
- What is the general perception of TB in your community? What do you yourself think about the disease?
- In your community, how is a person who has LTBI usually thought of?
- Will you feel comfortable to discuss your TB/latent TB diagnose and preventive treatment with other people in your community?
- In what ways may do you think it will be difficult to follow the advised recommendations regarding latent tuberculosis treatment? How do you think patients in general follow the recommendations?

**Evaluation of the film**

- What changes to this film would you suggest in order to improve its message?
- What did you think of the length of the film?
- Where and when do you think is the best location and timing to watch the film?
- What other means of information would you suggest to increase knowledge of TB?
- How do you usually get information about health issues and diseases?

“Tell me more…” “What do you mean?…” Can you give some examples?…” “How do you feel…?” “By this, do you mean…?”

Remember to be quiet more!

4. End of interview:

Do you have any questions to ask?

Ensure that the interviewee has full name and contact details of interviewer.

Thank the interviewee for their time!
