# Supplementary material for: Understanding tuberculosis among people with tuberculosis through an educational film: a qualitative study
Source: BMJ Open. 2025 Aug 19;15(8):e103199. doi: 10.1136/bmjopen-2025-103199 (PMC12366580; doi:10.1136/bmjopen-2025-103199)
Supplement: Supplementary file 3 [file bmjopen-2025-103199.reviewer_comments.pdf]

PEER REVIEW HISTORY

BMJ Open publishes all reviews undertaken for accepted manuscripts. Reviewers are asked to complete a checklist review form and are provided with free text boxes to elaborate on their assessment. These free text comments are reproduced below.

ARTICLE DETAILS

Title (Provisional)

Understanding tuberculosis among people with tuberculosis through an educational film: a qualitative study

Authors

Economou Lundeberg, Elin; Biermann, Olivia; Kuhlin, Johanna; Kulane, Asli; Annerstedt, Kristi Sidney; Davies Forsman, Lina

---

VERSION 1 - REVIEW

**Reviewer**	**1**
**Name**	**Rosnani, Zakaria**
**Affiliation**	**University Sains Malaysia - Health Campus, Department of Family Medicine**
**Date**	**14-May-2025**
**COI**	**None**

---

1. Abstract- semi structured questionnaires is a tool, the method was in depth interview

2. Strengths and limitations

- visual educational method (TB specimen displays, x-rays etc) for TB disease has been done however in non TB infection participants- as to my knowledge for e.g Idris NA, Zakaria R, Muhamad R, Nik Husain NR, Ishak A, Wan Mohammad WMZ. The Effectiveness of Tuberculosis Education Programme in Kelantan, Malaysia on Knowledge, Attitude, Practice and Stigma Towards Tuberculosis among Adolescents. Malays J Med Sci. 2020;27(6):102-114. doi:10.21315/mjms2020.27.6.10

- Limitation- only 13 participants and non-homogenous- 1 participant with TB disease and 12 participants with TB infection.

The manuscript acknowledges participants with varied experiences, which may enrich the data. However, it is important to also consider how these differences might influence their interpretation of the intervention and subsequently affect the consistency or credibility of the findings.

I recommend that the authors reflect on how participant heterogeneity was managed during analysis—for instance, through stratification, subgroup analysis, or reflexivity—in order to maintain analytical rigour. Addressing this would enhance the trustworthiness of the results and provide reassurance regarding the coherence of interpretations across differing experiential backgrounds.

Methodology

1. Theory- to explain further on the qualitative theory used in this study.

2. Framework- The manuscript mentions the use of a framework by Nuttall et al. to guide the identification of themes: “Themes were identified from associations between the categories and their meaning, and in relation to the framework.”

While this implies a framework-based thematic analysis (possibly deductive or theory-driven), the methodology section would benefit from clearer elaboration. Specifically, the authors should clarify:

How the framework by Nuttall et al. was applied in the data analysis process (e.g. during coding, theme development, or interpretation).

Whether themes were also allowed to emerge inductively from the data (i.e. a hybrid approach).

How the categories and associations were constructed and validated.

This clarification would strengthen the methodological rigour of the qualitative analysis and enhance transparency for readers unfamiliar with the cited framework.

3. Tool used- The authors mention the use of semi-structured questionnaires in data collection. However, further details would strengthen the transparency and reproducibility of the study.

Specifically, it would be helpful if the authors could elaborate on:

Whether there were any expansions or modifications to the initial set of questions during the interviews;

What prompted these expansions (e.g., participant responses, emerging themes, or contextual relevance); and

How such adaptations were handled to ensure consistency and rigour in data collection across participants.

Providing this clarification would enhance the methodological robustness and allow readers to better understand the flexibility and responsiveness of the data collection process.

4. The manuscript states that three researchers conducted the in-depth interviews (IDIs). Given the involvement of multiple interviewers, it would be important to clarify what steps,

if any, were taken to standardise the interview process and minimise potential interviewer bias.

For instance, were the researchers trained using a common protocol? Were mock interviews conducted, or were there any calibration sessions to align interview styles and questioning techniques?

Detailing such measures will enhance the credibility of the data collection process and reassure readers of the consistency and neutrality in how data were gathered across different participants.

Discussion-

The discussion would benefit from being more clearly anchored to the theoretical framework referenced in the study (e.g., Nuttall et al.). While the findings are described in detail, the manuscript should further explore how the results align with, extend, or challenge the theoretical constructs which supported the study.

A more detail integration of the theory into the interpretation of findings will strengthen the conceptual depth of the discussion and demonstrate the study's contribution to existing theoretical knowledge.

Conclusion- to revise in accordance with the title and objectives of the study.

Reviewer **2**

Name **Adisa, Rasaq**

Affiliation **University of Ibadan Faculty of Pharmacy, Clinical Pharmacy and Pharmacy Administration**

Date **03-Jun-2025**

COI **None**

As highlighted in the attached document

Thank you for the opportunity to review the above titled paper. Kindly find below my general and specific comments on the manuscript.

General

- **Originality** -Yes, the qualitative study was original with the use of an educational film, as an approach to educate and enlighten people living with TB about TB infection and the TB disease itself. The findings of the study contributed to the existing knowledge on newer approach for bridging the knowledge gaps, as well as increasing understanding about TB symptoms, transmission and treatment.

- **Importance of work to readers**: The study findings will further add to existing intervention approach that could be adopted when trying to sensitize and increase

awareness among people living with TB by the major stakeholders in TB care including researchers, clinicians, pharmacists. Disease association, teachers as well as other policymakers.

- **Evidence-based medicine**: The evidence provided in the study follows a standard reporting guideline except the small sample size of the participants (only 13 participants) in the study, which may prevent the generalization of the research findings to the entire TB population in the region.

- **Research question**: Although, the study did not specifically state any research question in the write-up, however, the specific aims/objectives of the study as highlighted in line 30-37 were appropriately answered with the study findings reported.

- **Study design**: The qualitative design guided by semi-structured interviews, and backed by the theoretical framework of Nutall et al employed in the study is appropriate and adequate for the conduct of the study.

- **Participants:** The study participants were adequately described, most especially the participants' demographic characteristics, however, the number of participants involved in the study seems too small and may not be representative of the eligible sample. Though, the authors mentioned the use of five dimensions of information power to estimate the study sample size.

- **Methods**: The study methods were elaborately and adequately described in the write up, with ethical considerations appropriately followed in participants' recruitment

- **Results**: The results of the thematic analysis from the verbatim patient's responses were concisely reported in line with the identified themes of the study. The results were well presented, credible and able to answer the study core objectives.

- **Interpretation and conclusions**: The study findings were succinctly discussed with relevant literature provided to support the findings. The explanation provided on the results interpretation is clear and largely focused on the precise findings from the study.

- **References:** The references provided are relevant, and up-to-date considering the fact that the study has been conducted since 2022. The lighter questions to ask the authors may be why the delay till 2025 before presenting the article for publication.

- **Abstract/summary/key messages**: Yes, the abstract accurately reflect what the study explored. However, there is need for a re-check of occasional grammatical error possibly by the native English speaker.

- **Reporting standards**: Yes, the study followed the COREQ criteria for reporting qualitative research and the checklist to ascertain such was provided by the authors.

Specific comments

Introduction

Line 9/10: …….. people with TB infection lacks clinical symptoms and are not contagious…… This statement may not be completely correct, more so author did not substantiate the statement with any relevant reference(s). Thus, a rephrase may be needed.

Line 14/15: ……….as the preventative regime, is shorter and has fewer side effects compared to TB disease treatment…… First, regime should be corrected as regimen. My question is what are those preventative regimens that has less side effects? Are they not also anti-TB drugs? Authors should have mentioned few of such preventive regimen so as to drive home the point.

Line 22/23: ………… currently only 47-76% globally ………. My comment is that a percentage of 47-76 is a huge figure and cannot be referred to as only.

Methods

Line 35/36 – Aim and design: …..to improve a possible later implementation ……….. This statement is unclear. It should be rechecked for grammatical error

Study setting and recruitment: Although, authors mentioned the use of five dimensions of information power in estimating the study sample size. But to the best of my knowledge, a study sample size of 13 participants overall is still very small for a representative sample size. Thus, generalization of the study findings to the entire population of TB may not be scientifically viable.

Data collection and analysis

Line 52-54: …………….I understand that a native English speaker was partly engaged for the translation of quotes in Swedish to English. I also believe the service of such native English speaker is necessary to proofread the entire manuscript for ensuring corrections of the occasional grammatical errors.

Results: Well-reported and adequately captured the study findings in line with the specific objectives of the study.

Discussion: The section was concisely discussed with relevant literature cited to buttress the findings.

Conclusion: The study conclusion pointedly addresses the core study objectives, with implication for practice provided.

I am very grateful for the opportunity to review the manuscript

---

**Reviewer**	**3**
**Name**	**Koch, Anastazia**
**Affiliation**	**University of Cape Town**
**Date**	**03-Jun-2025**

COI **None**

It's been a pleasure to review the study entitled "Understanding tuberculosis among people with tuberculosis through an educational film: a qualitative study.". The authors have used scholarly methods to investigate the impact of showing people who are either infected with Mtb or have active TB an educational film about TB. The study setting, Swedish health centres, would treat a combination of Swedish born and immigrant populations for TB. The research team was able to interview people in both groups and while the sample size was relatively small, 13 interviews in total, the authors generated insightful data and conclusions. The key findings include that the educational TB film

While the study is rigorous in terms of methods applied, is ethically sound and generates useful and relevant findings, I have recommended major revision for what is a minor, yet important issue. The authors did not include a link to the film in the study. In order to assess interviewee responses to the f

---

VERSION 1 - AUTHOR RESPONSE

Reviewer: 1

Dr. Zakaria Rosnani, University Sains Malaysia - Health Campus

We would like to express our sincere gratitude for your very insightful and helpful comments that have helped us improve the manuscript significantly.
Comments to the Author:
1. **Abstract**- semi structured questionnaires is a tool, the method was in depth interview

Thank you for pointing this out! We have now changed the first line in the "Method"-section of the Abstract. The sentence now reads:

Line 24: "We conducted a qualitative study, with in depth interviews (n=13), at two Infectious Disease Outpatient Departments in Sweden."*

We have also changed from "semi-structured interviews" to "in depth interviews" in the first sentence of the "Aim and design" section.

2. **Strengths and limitations**
- visual educational method (TB specimen displays, x-rays etc) for TB disease has been done however in non TB infection participants- as to my knowledge for e.g Idris NA, Zakaria R, Muhamad R, Nik Husain NR, Ishak A, Wan Mohammad WMZ. The Effectiveness of Tuberculosis Education Programme in Kelantan, Malaysia on Knowledge, Attitude, Practice and Stigma Towards Tuberculosis among Adolescents. Malays J Med Sci. 2020;27(6):102-114. doi:10.21315/mjms2020.27.6.10

Thank you for providing this reference, and we agree that visual educational material have been produced previously – for example the study you mention, and also a study by Spruijt et al. in the Netherlands (1). The visual material in these mentioned studies is in the lecture format, for example using pictures and Power Point-presentations. However, to our knowledge, few studies

have attempted to introduce a film. We have now specified in the bullet point that it is not "visual" per se that we mean, instead it is visual information in film format more specifically. The bullet point now reads:

"To the best of our knowledge, attempts of exploring the introduction of information about TB in the format of a film have rarely been done."

- Limitation- only 13 participants and non-homogenous- 1 participant with TB disease and 12 participants with TB infection.
The manuscript acknowledges participants with varied experiences, which may enrich the data. However, it is important to also consider how these differences might influence their interpretation of the intervention and subsequently affect the consistency or credibility of the findings. I recommend that the authors reflect on how participant heterogeneity was managed during analysis—for instance, through stratification, subgroup analysis, or reflexivity—in order to maintain analytical rigour. Addressing this would enhance the trustworthiness of the results and provide reassurance regarding the coherence of interpretations across differing experiential backgrounds.

We appreciate the reviewer's insightful comment regarding the heterogeneity of the participant sample and the potential implications for the credibility of our findings. As noted, our sample included one participant with TB disease and twelve with TB infection, representing a range of lived experiences with the condition.

In response to this, we have expanded our Strengths and Limitation section in the manuscript to explicitly reflect on how we accounted for this variation during the analysis. Specifically, we employed a reflexive thematic analysis approach that was attentive to differences in participant experiences. During coding and theme development, we consistently considered whether themes were shared across the spectrum of experiences or uniquely shaped by participants' TB status. Where relevant, we noted variations in how the intervention was interpreted by participants with TB disease versus infection. However, given the small sample size, formal subgroup analysis was not feasible. In addition, we believe that the detailed descriptions of our study population in the methods section increase the transferability of the findings and enhance their trustworthiness. Additional sentences in Strength and Limitations section now read:

"As a research team, we maintained reflexive awareness of the sample's heterogeneity, especially the inclusion of both individuals with TB infection and those with active TB disease. This awareness informed our coding and theme development, encouraging us to question whether our interpretations were shared or contingent upon participants' experiences with TB infection or disease. The team discussed our own assumptions about the potential influence of clinical background on perceptions of the intervention, and how this might have influenced our interpretation of the data. These reflexive practices supported a more nuanced and transparent analysis, which was crucial given the heterogeneity in our sample."

"The inclusion of only one participant with TB disease limited the depth of insight into how individuals with differing clinical presentations may interpret the intervention. While this limits transferability to all populations affected by TB, we believe the diversity of experiences within the infection group still provides meaningful insight into how the intervention may be received and adapted in practice."

Methodology

1. Theory – to explain further on the qualitative theory used in this study.

We thank the reviewer for this helpful comment regarding the theoretical underpinnings of our study. In response, we have expanded the Methods section to explicitly state the epistemological and theoretical orientation that guided the research. Specifically, we clarify that the study was informed by a constructionist epistemology, in which participant accounts are viewed as co-constructed and contextually embedded. We also describe how the conceptual model by Nuttall et al. served as a theoretical scaffold supporting our interpretation of how participants engaged with the intervention and made sense of their experiences.

New section "Theoretical underpinnings and frameworks": "This study was informed by a constructionist epistemology (2). We approached participant narratives not as objective accounts of reality, but as lived experiences that are situated and shaped by social, cultural, and personal factors. Our analysis was also guided by the conceptual model developed by Nuttall et al. (3), which offers a framework for understanding patient engagement and behavior in the context of TB care and specifically reducing TB-related stigma. We used this framework as a theoretical scaffold supporting our interpretation of how participants made sense of their TB experiences and the intervention, while remaining open to emergent themes beyond the scope of the model."

2. **Framework**- The manuscript mentions the use of a framework by Nuttall et al. to guide the identification of themes: "Themes were identified from associations between the categories and their meaning, and in relation to the framework."
While this implies a framework-based thematic analysis (possibly deductive or theory-driven), the methodology section would benefit from clearer elaboration. Specifically, the authors should clarify:
How the framework by Nuttall et al. was applied in the data analysis process (e.g. during coding, theme development, or interpretation).
Whether themes were also allowed to emerge inductively from the data (i.e. a hybrid approach).
How the categories and associations were constructed and validated.
This clarification would strengthen the methodological rigour of the qualitative analysis and enhance transparency for readers unfamiliar with the cited framework.

Thank you for your feedback. Predominately, we used an inductive approach in the data analysis process. The categories and themes were inductively derived from the data. Furthermore, while interpreting and presenting the themes we used the framework by Nuttall et al. as an aid. The themes are presented in the same order as Nuttall et al. have suggested in their framework, i.e. how stigma may diminish by different interventions. The categories thus emerged from the data, and they were validated by having the entire research group go through the codes and categories and together develop themes. We have clarified the abovementioned features of the data analysis process, and this section now reads:

Data collection and analysis, line 5: "From the codes, categories were inductively composed from patterns of meaning in the data and discussed with LDF and OB. Thereafter, categories were diagrammed into a concept map to find possible associations between the categories, and*

between their meanings. The categories were also related to the framework by Nuttall et al (3), by comparing the categories to the mechanisms of stigma reduction proposed by the framework (Figure 1). Themes were identified from associations between the categories and their meaning, and in relation to the framework. Phase 4 and 5 (reviewing and defining themes) of Braun and Clarke's thematic analysis process was discussed and revised by all co-authors. Examples of the thematic analysis process are presented in Table 2."

3. **Tool used-** The authors mention the use of semi-structured questionnaires in data collection. However, further details would strengthen the transparency and reproducibility of the study. Specifically, it would be helpful if the authors could elaborate on:
Whether there were any expansions or modifications to the initial set of questions during the interviews;
What prompted these expansions (e.g., participant responses, emerging themes, or contextual relevance); and
How such adaptations were handled to ensure consistency and rigour in data collection across participants.
Providing this clarification would enhance the methodological robustness and allow readers to better understand the flexibility and responsiveness of the data collection process.

Thank you for your reflections and questions. We agree that we should have elaborated on this in the Methods section. The topic guide was revised in the middle of the study period by EEL and KSA. This revision was initiated by the interviews and had two aims: First, we tried to slightly simplify the language used in the topic guides. Second, experience qualitative researched KSA gave us feedback on transcriptions from the interviews, and suggested how to improve the questions further. We have added a few sentences about this in the manuscript and have now two Appendices; one with the first topic guide and one with the revised version. The added sentences are:

Line 47: "The topic guide was further revised by EEL and KSA in the middle of the study period (appendix 2). The revision was initiated by the interviewers as some questions did not allow reflexive responses from the participants. Rephrasing some questions allowed for a better approach to asking the question, for example expanding the question "What is the general perception of TB in your community?" to include "What do you yourself think about the disease?".

4. The manuscript states that three researchers conducted the in-depth interviews (IDIs). Given the involvement of multiple interviewers, it would be important to clarify what steps, if any, were taken to standardise the interview process and minimise potential interviewer bias.
For instance, were the researchers trained using a common protocol? Were mock interviews conducted, or were there any calibration sessions to align interview styles and questioning techniques?
Detailing such measures will enhance the credibility of the data collection process and reassure readers of the consistency and neutrality in how data were gathered across different participants.

Thank you for your feedback. All three researchers had several correspondences discussing the topic guide and interview technique. However, some interviewer bias may still withstand

considering different training of interviewers and different locations, which is something that we have mentioned in the limitations section. On the other hand, EEL conducted nine out of 13 interviews which implies standardisation of the majority of interviews. We agree with your feedback and have now added a sentence about multiple interviewers in the Methods section:

Data collection and analysis, line 42: "As multiple interviewers conducted the interviews, all three interviewers were involved in the development of the topic guide, and discussed interview technique, before the first interview."

Discussion-
The discussion would benefit from being more clearly anchored to the theoretical framework referenced in the study (e.g., Nuttall et al.). While the findings are described in detail, the manuscript should further explore how the results align with, extend, or challenge the theoretical constructs which supported the study.

A more detail integration of the theory into the interpretation of findings will strengthen the conceptual depth of the discussion and demonstrate the study's contribution to existing theoretical knowledge.

We thank the reviewer for this valuable suggestion to more deeply integrate the theoretical framework by Nuttall et al. into our discussion. In response, we have revised the discussion section of the manuscript to explicitly relate our findings to key constructs in the framework, demonstrating where our data confirm, extend, or challenge these ideas. This expanded discussion aims to enhance the conceptual depth of the paper and clearly position our contribution within the existing theoretical literature on TB care engagement. Please see tracked changes in the manuscript.

Conclusion- to revise in accordance with the title and objectives of the study.

In line with changing the discussion we have now integrated the theoretical framework also to the conclusion and revised it accordingly. The conclusion now reads:

"In our study, the educational film was perceived by people with TB to increase their understanding of TB symptoms, transmission routes and treatment. Increasing understanding of TB constitutes the intended outcome of intervention pathways to reduce stigma suggested by Nuttall et al.'s theoretical framework (3). The film also challenged misconceptions and fears related to TB, described as a mechanism of stigma reduction. Changes to the film, suggested by the participants, can further improve the film. Through a better understanding of TB we anticipate a decrease in stigma and a potential increase in adherence to treatment (3), effects that should be further investigated after implementation of the film. "

Reviewer: 2 (see attached)

Dr. Rasaq Adisa, University of Ibadan Faculty of Pharmacy
Comments to the Author:
As highlighted in the attached document

Thank you for the opportunity to review the above titled paper. Kindly find below my general and specific comments on the manuscript.

General

• **Originality** -Yes, the qualitative study was original with the use of an educational film, as an approach to educate and enlighten people living with TB about TB infection and the TB disease itself. The findings of the study contributed to the existing knowledge on newer approach for bridging the knowledge gaps, as well as increasing understanding about TB symptoms, transmission and treatment.

Thank you for your kind words and well-structured feedback!

• **Importance of work to readers:** The study findings will further add to existing intervention approach that could be adopted when trying to sensitize and increase awareness among people living with TB by the major stakeholders in TB care including researchers, clinicians, pharmacists. Disease association, teachers as well as other policymakers.

• **Evidence-based medicine**: The evidence provided in the study follows a standard reporting guideline except the small sample size of the participants (only 13 participants) in the study, which may prevent the generalization of the research findings to the entire TB population in the region.

The sample size has been estimated via the method of "information power" by Malterud et al. Through the five dimensions of information power (Aim, Specificity, Theory, Dialogue, Analysis) we concluded that 13 participants would suffice to get enough information from our participants. Furthermore, throughout the interview process we considered adding more participants, but concluded that it would not add any new information. We are making the study results transferable, the aim of qualitative research compared to generalisable in quantitative methodology, by describing thoroughly the study setting and being transparent about the analysis process (4).

• **Research question:** Although, the study did not specifically state any research question in the write-up, however, the specific aims/objectives of the study as highlighted in line 30-37 were appropriately answered with the study findings reported.

• **Study design:** The qualitative design guided by semi-structured interviews, and backed by the theoretical framework of Nutall et al employed in the study is appropriate and adequate for the conduct of the study.

• **Participants:** The study participants were adequately described, most especially the participants' demographic characteristics, however, the number of participants involved in the study seems too small and may not be representative of the eligible sample. Though, the authors mentioned the use of five dimensions of information power to estimate the study sample size.

Regarding the sample size, please see answer above, after "Evidence-based medicine".

• **Methods:** The study methods were elaborately and adequately described in the write up, with ethical considerations appropriately followed in participants' recruitment

• **Results:** The results of the thematic analysis from the verbatim patient's responses were concisely reported in line with the identified themes of the study. The results were well presented, credible and able to answer the study core objectives.

• **Interpretation and conclusions:** The study findings were succinctly discussed with relevant literature provided to support the findings. The explanation provided on the results interpretation is clear and largely focused on the precise findings from the study.

• **References:** The references provided are relevant, and up-to-date considering the fact that the study has been conducted since 2022. The lighter questions to ask the authors may be why the delay till 2025 before presenting the article for publication.

The interviews were conducted alongside clinical work for the three infectious disease physicians involved in the study. Consequently, it took more than a year to conduct the interviews. Furthermore, the corresponding author being a part-time PhD-student, involved a perceivably longer period of time to complete the analysis process and manuscript writing.

• **Abstract/summary/key messages:** Yes, the abstract accurately reflects what the study explored. However, there is need for a re-check of occasional grammatical error possibly by the native English speaker.

Thank you for the advice. A native English speaker has now proof-read the manuscript.

• **Reporting standards:** Yes, the study followed the COREQ criteria for reporting qualitative research and the checklist to ascertain such was provided by the authors.

Specific comments

Introduction

Line 9/10: .... people with TB infection lacks clinical symptoms and are not contagious...... This statement may not be completely correct, more so author did not substantiate the statement with any relevant reference(s). Thus, a rephrase may be needed.

Thank you for your suggestion. Even though tuberculosis is more and more acknowledged as a spectrum of disease presentations, TB infection is still considered, to our knowledge, to be non-contagious and without symptoms for the individual. However, we completely agree that a reference should be added, and we have done so accordingly (Latent tuberculosis infection: updated and consolidated guidelines for programmatic management. Geneva: World Health Organization; 2018).

Line 14/15: ....as the preventative regime, is shorter and has fewer side effects compared to TB disease treatment...... First, regime should be corrected as regimen. My question is what are those preventative regimens that has less side effects? Are they not also anti-TB drugs? Authors should have mentioned few of such preventive regimen so as to drive home the point.

Thank you for pointing this out! We have now changed the word "regime" to regimen, and we have also revised the sentence to make it clearer. The point we were trying to make was that the preventative regimens usually contain fewer drugs, and are completed quicker, than TB disease regimens. The sentence now reads:

Line 14: "Preventative treatment of TB infection is preferable, as the preventative regimens are shorter and contains fewer drugs compared to TB disease treatment."

Line 22/23: .... currently only 47-76% globally .... My comment is that a percentage of 47-76 is a huge figure and cannot be referred to as only.

Thank you for highlighting this and we agree that the numbers need to be specified. We have now added the settings of the studies, and separated the numbers. The sentence now reads:

Line 22: "However, adherence to treatment of TB infection can be as low as 47 %, shown in a study from the US and Canada (5), or 76 % in Swedish setting (6)."

Methods

Line 35/36 – Aim and design: .....to improve a possible later implementation .... This statement is unclear. It should be rechecked for grammatical error.

We have tried to clarify this sentence by adding that it is the film that we could improve before implementing the film in Swedish/Nordic outpatient departments. The sentence now reads:

Line 35: "Second, we aimed to collate the patients' views regarding the educational film, in order to improve it before a possible future implementation."

Study setting and recruitment: Although, authors mentioned the use of five dimensions of information power in estimating the study sample size. But to the best of my knowledge, a study sample size of 13 participants overall is still very small for a representative sample size. Thus, generalization of the study findings to the entire population of TB may not be scientifically viable.

We thank the reviewer for their comment and the opportunity to clarify the methodological foundations of qualitative research. As noted, this study applied the concept of information power (Malterud et al., 2016) to guide sample adequacy, which is consistent with established practices in qualitative inquiry. In qualitative research, the goal is not to achieve statistical representativeness or generalizability, but rather to generate in-depth, contextually rich insights into the experiences and meanings constructed by participants.

We have therefore revised the manuscript slightly to clarify this distinction, emphasizing that our findings are intended to be transferable to similar contexts rather than generalizable in a statistical sense. We hope this clarification addresses the concern and reinforces the appropriateness of the sample size and design within the qualitative paradigm.

Strength and limitations section, added sentences: "While this limits transferability to all populations affected by TB, we believe the diversity of experiences within the infection group still provides meaningful insight into how the intervention may be received and adapted in practice."

Data collection and analysis

Line 52-54: ....I understand that a native English speaker was partly engaged for the translation of quotes in Swedish to English. I also believe the service of such native English speaker is necessary to proofread the entire manuscript for ensuring corrections of the occasional grammatical errors.

Thank you for the advice. A native English speaker has now proof-read the manuscript.

Results: Well-reported and adequately captured the study findings in line with the specific objectives of the study.

Discussion: The section was concisely discussed with relevant literature cited to buttress the findings.

Conclusion: The study conclusion pointedly addresses the core study objectives, with implication for practice provided.

I am very grateful for the opportunity to review the manuscript

Reviewer: 3

Dr. Anastazia Koch, University of Cape Town, EHWOZA
Comments to the Author:
It's been a pleasure to review the study entitled "Understanding tuberculosis among people with tuberculosis through an educational film: a qualitative study.". The authors have used scholarly methods to investigate the impact of showing people who are either infected with Mtb or have active TB an educational film about TB. The study setting, Swedish health centres, would treat a combination of Swedish born and immigrant populations for TB. The research team was able to interview people in both groups and while the sample size was relatively small, 13 interviews in total, the authors generated insightful data and conclusions. The key findings include that the educational TB film

While the study is rigorous in terms of methods applied, is ethically sound and generates useful and relevant findings, I have recommended major revision for what is a minor, yet important issue. The authors did not include a link to the film in the study. In order to assess interviewee responses to the f

Thank you for reading our article and your kind words! The final comment seems to be cut off, and we have emailed the Editor's Office regarding this. The films are available, with open access, online: [Tuberkulos - Vårdprogram från SILF - Infektion.net](). We have now included this information in the manuscript under the section "Data Availability Statement".

References

1. Spruijt I, Haile DT, van den Hof S, Fiekert K, Jansen N, Jerene D, et al. Knowledge, attitudes, beliefs, and stigma related to latent tuberculosis infection: a qualitative study among Eritreans in the Netherlands. BMC Public Health. 2020;20(1):1602.
2. Green JT, N. Qualitative methods for Health Research. 4 ed: SAGE; 2018.
3. Nuttall C, Fuady A, Nuttall H, Dixit K, Mansyur M, Wingfield T. Interventions pathways to reduce tuberculosis-related stigma: a literature review and conceptual framework. Infect Dis Poverty. 2022;11(1).
4. Nowell LS, Norris JM, White DE, Moules NJ. Thematic Analysis:Striving to Meet the Trustworthiness Criteria. Int J Qual Methods. 2017;16(1):1609406917733847.
5. Horsburgh CR, Goldberg S, Bethel J, Chen S, Colson PW, Hirsch-Moverman Y, et al. Latent TB Infection Treatment Acceptance and Completion in the United States and Canada. Chest. 2010;137(2):401-9.
6. Kan B, Kalin M, Bruchfeld J. Completing treatment for latent tuberculosis: patient background matters. Int J Tuberc Lung Dis. 2013;17(5):597-602.

VERSION 2 - REVIEW

**Reviewer**	**1**
**Name**	**Rosnani, Zakaria**

Affiliation **University Sains Malaysia - Health Campus, Department of Family Medicine**

Date **06-Jul-2025**

COI

Good job

The manuscript is more understandable now

However just a minor improvement needed

1. Result-

"However, the film challenged these fears and increased the understanding of TB infection being treatable and non-infectious"

Kindly used proper term which is latent TB infection or LTBI, because active TB is also TB infection.

To use proper term throughout the manuscript to avoid confusion with active TB.

2. Study setting and recruitment

"we decided to conduct 13 additional interviews."

To clarify how many interviews were conducted in this study? 13 additional interviews convey the meaning that they were interviews conducted before and then another 13 interviews were added.

VERSION 2 - AUTHOR RESPONSE

Reviewer: 1
Dr. Zakaria Rosnani, University Sains Malaysia - Health Campus
Comments to the Author:
Good job
The manuscript is more understandable now

Thank you!

However just a minor improvement needed
1. Result-
"However, the film challenged these fears and increased the understanding of TB infection being treatable and non-infectious"
Kindly used proper term which is latent TB infection or LTBI, because active TB is also TB infection.
To use proper term throughout the manuscript to avoid confusion with active TB.

Thank you for your valuable feedback. We understand the importance of using terminology that avoids confusion between active and non-active TB. However, in accordance with the 2024 WHO

consolidated guidelines on tuberculosis [1], the term "TB infection" is now used in place of "latent TB infection (LTBI)" to reflect the continuum between infection and disease.

To maintain clarity for all readers, we have revised the manuscript to include an explanatory note upon first mention of "TB infection" in the main text of the article, clarifying that this term aligns with WHO's updated nomenclature and was previously referred to as LTBI. We hope this approach addresses your concern while remaining consistent with current international guidelines.

The sentences in the manuscript now reads: "Individuals with TB infection (previously referred to as latent TB infection, LTBI) lack clinical symptoms but are at risk of progressing to active TB disease [1]"

2. Study setting and recruitment

"we decided to conduct 13 additional interviews."
To clarify how many interviews were conducted in this study? 13 additional interviews convey the meaning that they were interviews conducted before and then another 13 interviews were added.

Thank you for pointing this out! We completely agree that this wording leads to misinterpretation, and we have now clarified the number of interviews done.

The sentences now read: "Study participants were all highly specific to the target group, and together with an underlying theoretical framework, we originally estimated a sample size of around ten participants. However, the quality of the interview dialogues was varying and as an exploratory analytic approach was used, we decided to conduct an additional three interviews."

Reference

1. WHO consolidated guidelines on tuberculosis. Module 1: prevention – tuberculosis preventive treatment, second edition. Geneva: World Health Organization; 2024.
